# A Digital Sexual Health Education Web Application for Resource-Poor Regions in Kenya: Implementation-Oriented Case Study Using the Intercultural Research Model

**DOI:** 10.2196/58549

**Published:** 2024-07-03

**Authors:** Clarissa Soehnchen, Anja Burmann, Maike Henningsen, Sven Meister

**Affiliations:** 1 Health Informatics School of Medicine, Faculty of Health Witten/Herdecke University Witten Germany; 2 Department Healthcare Fraunhofer Institute for Software and Systems Engineering Dortmund Germany; 3 School of Medicine Faculty of Health Witten/Herdecke University Witten Germany

**Keywords:** sexual health education, Intercultural Research Model, semistructured interview, SUS analysis, user-centered design

## Abstract

**Background:**

Developing a digital educational application focused on sexual health education necessitates a framework that integrates cultural considerations effectively. Drawing from previous research, we identified the problem and essential requirements to incorporate cultural insights into the development of a solution.

**Objective:**

This study aims to explore the Solution Room of the self-established Intercultural Research Model, with a focus on creating a reusable framework for developing and implementing a widely accessible digital educational tool for sexual health. The study centers on advancing from a low-fidelity prototype (She!Masomo) to a high-fidelity prototype (We!Masomo), while evaluating its system usability through differentiation. This research contributes to the pursuit of Sustainable Development Goals 3, 4, and 5.

**Methods:**

The research methodology is anchored in the Solution Room of the self-expanded Intercultural Research Model, which integrates cultural considerations. It uses a multimethod, user-centered design thinking approach, focusing on extensive human involvement for the open web-based application. This includes gathering self-assessed textual user feedback, conducting a System Usability Scale (SUS) analysis, and conducting 4 face-to-face semistructured expert interviews, following COREQ (Consolidated Criteria for Reporting Qualitative Research) guidelines.

**Results:**

Based on the identified limitations of the low-fidelity prototype, She!Masomo (SUS score 67), which were highlighted through textual user feedback (63/77) and prototype feature comparisons, iterative development and improvement were implemented. This process led to the creation of an enhanced high-fidelity prototype (We!Masomo). The improved effectiveness of the enhanced prototype was evaluated using the qualitative SUS analysis (82/90), resulting in a favorable score of 77.3, compared with the previous SUS score of 67 for the low-fidelity prototype. Highlighting the importance of accessible digital educational tools, this study conducted 4 expert interviews (4/4) and reported e-survey results following the CHERRIES (Checklist for Reporting Results of Internet E-Surveys) guideline. The digital educational platform, We!Masomo, is specifically designed to promote universal and inclusive free access to information. Therefore, the developed high-fidelity prototype was implemented in Kenya.

**Conclusions:**

The primary outcome of this research provides a comprehensive exploration of utilizing a case study methodology to advance the development of digital educational web tools, particularly focusing on cultural sensitivity and sensitive educational subjects. It offers critical insights for effectively introducing such tools in regions with limited resources. Nonetheless, it is crucial to emphasize that the findings underscore the importance of integrating culture-specific components during the design phase. This highlights the necessity of conducting a thorough requirement engineering analysis and developing a low-fidelity prototype, followed by an SUS analysis. These measures are particularly critical when disseminating sensitive information, such as sexual health, through digital platforms.

**International Registered Report Identifier (IRRID):**

RR2-10.1186/s12905-023-02839-6

## Introduction

### Background

Sub-Saharan Africa has a population of approximately 1.1 billion people, representing roughly 14% of the global population. Despite a decrease in the global share of new HIV infections in the region, sub-Saharan Africa still accounted for 59% of all new infections worldwide in 2021 [1]. The region accounts for two-thirds of new sexually transmitted infection cases [2], and HIV continues to be the primary cause of death among adolescents in sub-Saharan Africa [3]. In 10 out of 11 countries in Western and Central Africa, less than half of the women surveyed reported being able to make their own decisions regarding sexual relations, contraceptive use, and their own health care [1]. Furthermore, over 80% of individuals report incidents of technology-facilitated sexual violence, gender-based violence, or other forms of violence [2,4]. Given these challenges in sub-Saharan Africa, it is imperative to reduce unplanned pregnancies and disease transmission. This entails providing adolescents with access to contraception information [4] and comprehensive sexual health education to help them make informed decisions. Access to knowledge empowers individuals to make sustainable choices regarding their bodies and health. Adolescents often lack access to crucial health care information [1]. One potential reason is the absence of comprehensive sex education in the national school curriculum, leaving teachers ill-equipped to address these topics in the classroom [5], particularly in Kenya. Kenya is selected as an example for this study due to its network of community centers, mobile infrastructure, and presence of low-income regions, which facilitate evaluation. The absence of structured, verified, valid, and reliable sexual information, particularly concerning contraception, menstruation, and female genital mutilation, leaves young women vulnerable to health risks [6]. Additionally, sexual health discussions are influenced by religious, tribal, and social affiliations, which can result in inconsistent or suppressed conversations [6,7]. The societal taboo surrounding contraceptive practices can drive some individuals to resort to fatal abortions to prevent unwanted pregnancies [8]. Given the challenges of limited infrastructure, financial resources, and access to education and sexual health information, the increased use of the internet and digital technologies presents an opportunity for promoting behavioral change [9]. The literature demonstrates a growing emphasis on digital tools to address these issues [6,8,10-15]. A study providing contraceptive information to young people [16] cites research indicating that mass media, particularly digital platforms, effectively reach and educate young adults about sexual health. Another study confirmed that adolescents in Kenya express a desire for easily accessible and trustworthy information about maintaining sexual health [17]. The use of digital tools and social media has bolstered confidence in addressing these topics, allowing for feedback and dispelling misconceptions [6,10,11]. Disseminating information can significantly reduce the risks of sexually transmitted infections such as HIV and unplanned pregnancies, with considerable potential for educating low-income and vulnerable communities [12,13]. However, ensuring lasting user engagement remains crucial [11].

Design thinking methodologies, as endorsed by Plattner et al [18], provide a creative framework for effectively addressing social and cultural challenges, specifically focusing on human needs. This involves collaborating with diverse groups, continually refining the process through interactions, and considering cultural-ergonomic factors to ensure the usability and accessibility of the final product [19-21]. The Double Diamond Model forms the foundation of the design thinking approach, providing a structured process; however, it typically lacks integration of intercultural principles. In the realm of design thinking processes, recognizing the significance of cultural consistency, as underscored by Kroeber and Kluckhohn [20], is essential for developing digital educational tools aimed at achieving cross-cultural usability.

### The Intercultural Research Model

**Figure 1:**
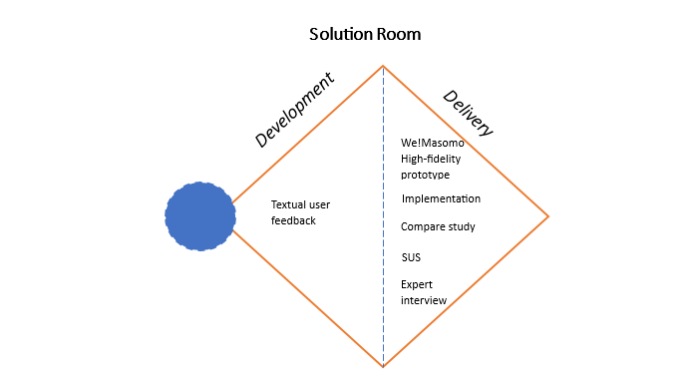
Case study design and analysis steps. SUS: System Usability Scale.

In a prior study by the author, the original Double Diamond Model (see Figure S1 in Multimedia Appendix 1) was adapted to the Intercultural Research Model, depicted in Figure 1, to identify requirements and design principles, enhancing focus on user needs [22]. Hence, the research model was enriched with cultural considerations, drawing insights from Rau et al [23], Lachner et al [24], and Laws et al [25], culminating in the development of a solution as depicted in Figure 1. The model empowers users from specific cultures to engage with products developed for foreign cultures, thereby enhancing accessibility and usability on a broader scale in the future. It establishes connections between diverse cultural spheres through varied perspectives. In the context of globalization, this process becomes imperative [26]. When addressing sexual health education in Kenya, it is essential to explore diverse perspectives beyond traditional literature to foster innovation. Culture plays a pivotal role in shaping both unconscious and conscious behaviors within social groups, influencing decision-making processes and various characteristics [20]. First, the Problem Room, divided into discover and define phases [24], conducts requirement engineering analysis. In phase 1, the discover phase identifies user requirements, defining primary, secondary, and anti-persona, alongside conducting empathy maps and storyboards [15]. Phase 2, the define phase, conceptualizes a cross-cultural design philosophy. Focusing on user, environment, and cultural components, based on Smith-Jackson et al’s [27] cultural ergonomics, results in a requirement engineering analysis (see Table S1 in Multimedia Appendix 1; also see [7,28]) [22,29]. In phase 3, the development phase meticulously outlines the product, integrating cultural and end user needs, while creating a low-fidelity prototype using a user interface/user experience design tool. The delivery phase, phase 4, encompasses high-fidelity prototype development, implementation, and delivery to the end user, building on the initial 3 phases [22]. Refer to Figure S2 in Multimedia Appendix 1 for the Intercultural Research Model, which is an adaptation based on the Nielsen Norman Group’s Double Diamond Model and insights from Soehnchen et al [22,29] and Rau et al [23].

### From She!Masomo to We!Masomo

The initial phase of the Intercultural Research Model involves conducting a requirement engineering analysis, which provides the foundation for developing a low-fidelity prototype. In this development process, the low-fidelity prototype, named She!Masomo, is specifically designed to focus on women’s sexual health education [22]. The prototype, named She!Masomo, was developed using Balsamiq Software [30] to visualize requirements and demonstrate user interactions aligned with Kenyan culture and educational knowledge status. It underwent an end user Unified Theory of Acceptance and Use of Technology and System Usability Scale (SUS) evaluation, collecting feedback and improvement suggestions for the final high-fidelity prototype as part of the development phase in the Solution Room [29], depicted in Figure 1. As a result, collected end user feedback was implemented to improve the development of the high-fidelity prototype, leading to the rebranding from She!Masomo to We!Masomo. We!Masomo is an all-gender inclusive web-based application for sexual health education, integrating She!Masomo, represented by its fictional character, Linda, and He!Masomo, featuring its fictional character Leo.

### Study Objectives

This paper focuses on the Solution Room, encompassing the missing parts of the development and the not-yet-addressed delivery phases, culminating in the high-fidelity prototype—the digital educational tool named We!Masomo. The objective of We!Masomo is to provide free digital information on contraceptive methods and menstruation education to empower and educate users. Given that the targeted end users have diverse cultural backgrounds, their inclusion in the process is imperative. Societal stigmas, cultural and religious backgrounds, and misconceptions [7,8,28] are identified as barriers to sexual health knowledge. However, acceptance depends on various factors, and improving sexual health is deemed a major necessity [11], highlighting the need for tailored educational content to address specific circumstances. Recognizing the impact of cultural differences on product usability, Barber and Badre [31] encapsulate this concept as “culturability.” Consequently, this research aligns with cultural ergonomics, as outlined by Smith-Jackson et al [27], integrating culture into the development process of designing human-computer interaction systems. The purpose of this case study is to design and implement We!Masomo in a way that addresses the challenges of limited access to knowledge, especially in the field of sexual education. Therefore, our research question is “To what extent can the Intercultural Research Model support the development and delivery phases (Solution Room) with a focus on creating a high-fidelity prototype?” This question considers the following objectives:

- Objective 1: To evaluate system usability as a measure of the fit between the low-fidelity and high-fidelity prototypes during the transition from the Problem Room to the Solution Room.
- Objective 2: To differentiate between She!Masomo and the newly developed all-inclusive platform We!Masomo.

We hypothesize that not all established requirements can be fully met during the development of the high-fidelity prototype and may indicate levels of digital literacy. However, we posit that this shortfall will not negatively impact the measurement of system usability.

## Methods

### Case Study Approach Within the Development and Delivery Phases

Case study research is historically closely associated with the methodology outlined by Yin [32], encompassing preparation, data collection, analysis, and reporting. This approach is well-suited for investigating contemporary phenomena within the natural context of software engineering. Runeson and Höst [33] provide guidelines for conducting and reporting case study research in software engineering. They emphasize the importance of understanding what constitutes a case study and its real-life context, specifically in the case of We!Masomo. The authors compiled terminology and guidelines from various methodology handbooks in the social sciences and information systems fields, adapting them to meet the specific needs of software engineering. They stress the significance of checklists for researchers and readers, derived through a systematic analysis of existing literature [34]. The case study methodology is well suited for various types of software engineering research, as it focuses on contemporary phenomena that are difficult to study in isolation. This case study aligns with the process suggested by Runeson and Höst [33], which includes case study design, preparation for data collection, data collection, analysis of collected data, and reporting.

### Case Study Design Within the Development and Delivery Phases: From the Low-Fidelity Prototype She!Masomo to Developing the High-Fidelity Prototype We!Masomo

The development phase of the Solution Room resulted in the creation of a low-fidelity prototype, She!Masomo, which analyzed the end user’s environment. This prototype visually represented user requirements and was demonstrated through abstract depictions in established storyboards [24,32]. In the subsequent delivery phase, the focus shifts to the high-fidelity prototype, now named We!Masomo, designed for sexual health education in sub-Saharan Africa. This evolution includes expanding content and broadening the target group to encompass both women and men. Our case study approach aims to be holistic, focusing on investigating and describing the transition from She!Masomo to We!Masomo, with attention to the objectives outlined in the introduction. The scope is confirmatory and explanatory, aiming to enhance the user experience of We!Masomo. Data and method triangulation are employed using a mixed methods approach, structured in the steps outlined in Textbox 1.

**Steps in the mixed methods approach applied in this study.**

1. Step 1
  - Conduct a descriptive analysis of textual user feedback by evaluating descriptive feedback collected from the low-fidelity prototype, She!Masomo.
  - Identify improvement suggestions derived from end user evaluation and recommend enhancements.
2. Step 2
  - Conduct a prototype comparison by comparing the previously established low-fidelity click-dummy, She!Masomo, with the high-fidelity prototype, We!Masomo.
  - Highlight eliminated or adjusted requirements during the development process.
3. Step 3
  - Conduct a System Usability Scale (SUS) analysis for the We!Masomo web application.
  - Utilize the widely recognized qualitative SUS questionnaire to assess the usability and user perception of the high-fidelity prototype (We!Masomo).
  - Apply descriptive statistics for a product-centered evaluation of SUS analysis to quantify the usability of the software product [35].
  - Compare SUS analysis results with those of She!Masomo, analyzed in the development phase [29].
  - Follow this with improvement suggestions derived from end user evaluation and enhancement recommendations for We!Masomo, highlighting the evolution of requirements during development.
4. Step 4
  - Conduct qualitative semistructured expert interviews with local supervisors of potential end users.
  - Explore insights and gather long-term improvement suggestions using semistructured questions designed to facilitate open discussion and diverse feedback.
  - Align the interview approach with the 32-item checklist known as the COREQ (Consolidated Criteria for Reporting Qualitative Research) guidelines [36,37].

Each step focuses on a specific aspect of the evaluation process, providing a clear and structured presentation of the analysis conducted throughout the development process leading to the final product, the high-fidelity prototype, We!Masomo. Figure 1 illustrates these essential steps.

### Preparation for Data Collection and Recruiting

The preparation, recruitment, and collection of implementation user feedback data are divided into 3 distinct research phases to facilitate the subsequent 4-step analysis, in accordance with triangulation requirements.

The first research phase, which involved qualitative descriptive analysis of textual user feedback on She!Masomo, took place from December 1, 2022, to January 31, 2023. This phase was implemented through community centers, girls’ schools, and universities in rural regions of Kenya, as well as during a field trip. A convenience sampling technique was used to reach the predominantly female target group aged 18-35. To ensure participant support, the survey on the low-fidelity prototype was supplemented with in-person assistance from January 11 to January 23, 2023. Participants self-assessed through online questionnaires at various community centers in Eldoret (Making More Health, MOI University in Eldoret, and Learning Lions, each visited for 3 days). To ensure a comfortable environment for discussing the sensitive topic, the in-person support team, consisting of the main author and trusted local representatives, conducted a brief introduction to the tool and its mission. This was followed by a 20-minute testing session of the low-fidelity prototype using participants’ smartphones or provided smartphones. The survey was conducted using the online platform LimeSurvey (LimeSurvey Team), where all participants consented to participate, and no indication of unwillingness to participate in the study was detected [38]. The vulnerability of the target group is amplified by gendered social norms, practices, and inequalities, which are particularly pronounced in sub-Saharan Africa, where rates of child marriage are the highest globally [1].

The second research phase involved the SUS analysis of We!Masomo, conducted between November 15, 2023, and December 31, 2023. The online platform LimeSurvey was used, and the survey was distributed through community centers, high schools, and universities in both rural and urban areas of Kenya, using a convenience sampling technique. All participants provided consent, and no indication of unwillingness to participate in the study was detected [38]. The study targeted males and females aged 18-35 years. To ensure participant support and improve response rates, in-person survey facilitation took place from November 15 to December 15, 2023. This approach aimed to create a comfortable environment for discussing sensitive topics and addressing potential challenges related to comprehension of content and language. The web-based application enabled participants to access and review the provided content. After an exploration period, participants proceeded to complete the survey using their devices. For those without digital devices, smartphones or laptops were provided, allowing them a 20-minute window to explore the high-fidelity prototype through the web-based application. After exploration, participants completed the survey on the provided device.

The third data collection research phase involved qualitative expert interviews conducted by the author CS, who is female and the founder of We!Masomo. The interviews were recorded using Microsoft Teams for audio recording and coded by CS in alignment with COREQ guidelines [38]. As a result of data saturation, 4 expert interviews were deemed sufficient. All participants provided consent. No indication of unwillingness to participate during the interviews was detected [38]. Additionally, to ensure a supportive environment and address potential comprehension challenges, a local supervisor was present. The interviews had a duration ranging from 30 to 70 minutes [38].

### Data Analysis and Reporting

**Figure 2:**
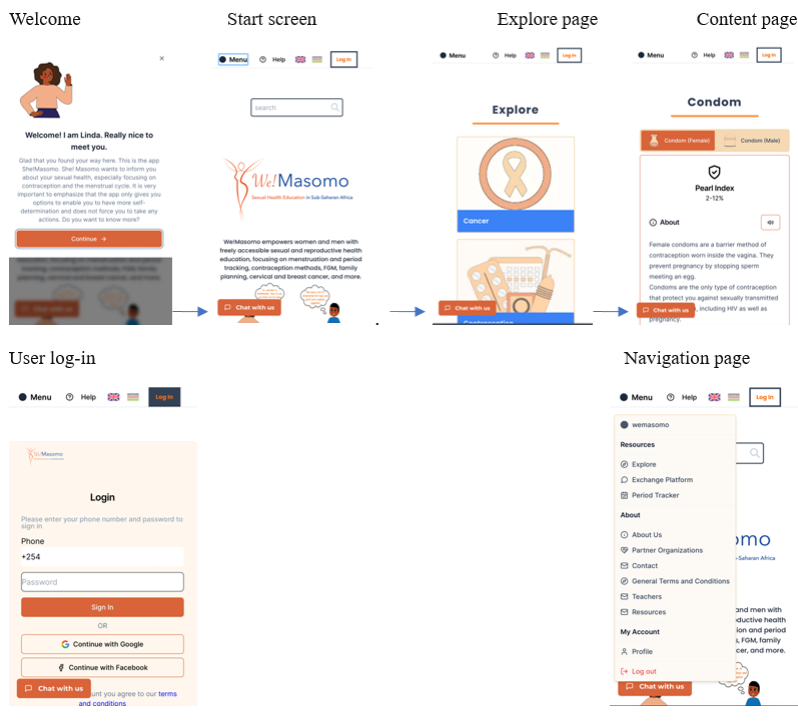
The Welcome screen when opening. Also shown are the introduction to the content and the mechanism of the application.

Based on our triangulation approach, the data analysis was divided into 4 steps: step 1 involved a descriptive analysis of collected textual user feedback on the low-fidelity prototype; step 2 entailed prototype comparison; step 3 involved conducting an SUS analysis on We!Masomo and comparing the SUS score with that of the low-fidelity prototype; step 4 consisted of qualitative expert interviews using qualitative research methods on the high-fidelity prototype, as illustrated in Figure 2.

The first step involved descriptive analysis of textual user feedback to characterize the sample of 77 study respondents. Feedback collected during the initial fieldwork study from December 1, 2022, to January 31, 2023, was analyzed using grounded theory [33,39]. Grounded theory facilitates systematic collection and categorization of textual user feedback related to the research questions. Each idea was noted and organized into thematic areas, which facilitated a comprehensive understanding [33]. Ideas, content, and quotes from each participant were individually written on notes, using different colors to distinguish thematic areas. This approach allowed for a nuanced analysis of feedback, considering its origin and facilitating inductive categorization. The clustering process, guided by predefined dimensions, also incorporated additional categories and subcategories, resulting in the identification of 4 main clusters with subcategories. The textual feedback was systematically allocated to these categories and subcategories. In response to user feedback and improvement suggestions, the development of the high-fidelity prototype culminates in the rebranding to We!Masomo.

Step 2 involved a comparative study of the low- and high-fidelity prototypes to evaluate the usability and features of We!Masomo. Features and content were systematically analyzed to identify enhancements and improvements in the high-fidelity version.

Step 3 involved a qualitative SUS analysis of We!Masomo. The online SUS analysis, a quantitative method, assessed usability divided into 2 sections. Section A gathered demographic data and information on respondents’ patterns of electronic device usage for accessing health-related information. Section B incorporated the standardized SUS questionnaire. The SUS questionnaire consists of 10 items rated on a 5-point Likert scale, yielding scores between 0 and 100 [40]. Scores of 68 or above indicate good usability [40,41]. For reporting e-survey results, the recommended CHERRIES (Checklist for Reporting Results of Internet E-Surveys) checklist by Eysenbach [37] was followed (see Multimedia Appendix 2 and Table 1).

**Table 1:** Feature comparison of low- versus high-fidelity prototype.

Feature	Low-fidelity prototype	High-fidelity prototype
Explore page	Content: Information on menstruation, contraception methods, 15 female genital mutilation videos, potential list of health care centers, partnership organizations, about She!Masomo	Added content: Cervical cancer, breast cancer, mental health issues, parenting content, exchange platform, He!Masomo for male-focused content, period tracking option, Leo and Linda as comic figures, and extended user profile option
Landing page	African Map, Linda is shown, She!Masomo logo	Changed color scheme, changed the logo to We!Masomo, directed to the “Explore” page, cookie request, and introduction by Leo and Linda
Added content	Menstruation content, contraception methods, 15 female genital mutilation videos	Cervical cancer, breast cancer, parenting content, mental help support content, and Swahili audio files
Hotline	—^a^	Not yet—in discussion with a partner organization for emergency abuse cases
Gender	Female (Linda)	Male and female (Leo and Linda comic figures)
Free access to content	All content	Content of menstruation information, cervical and breast cancer, and contracepting methods
Logo	She!Masomo	We!Masomo—as an all-inclusive platform for She!Masomo and He!Masomo
User profiles	Not necessary	Added verified user profile via phone number or email address
Exchange platform	—	Added a platform so users can ask questions only via verified user profiles
Period tracker	—	Added a period tracking feature as a natural contraction option; can track mood and menstruation
Contact formula	—	Contact option for specific questions and feedback
Feedback option	—	Added a thumbs up/down option as a direct feedback option per provided content.
Languages	Swahili and English	Swahili and English audio files for the read aloud function
User profiles	—	General questions, family status, menstruation information, use of contraception method, female genital mutilation—besides the general information, filling out the rest is optional^b^

^a^Not applicable.

^b^When the user registers on the tool and creates a user profile, the user is not required to provide all information (ie, users are only required to provide certain information such as birth year, gender, and a creative username).

The fourth and final analysis step involved qualitative expert interviews using qualitative research methods. Expert interviews provide deep insights into user experiences, perceptions, and suggestions for improvement. This includes using one of Kuckartz’s [42] approaches for content-structuring qualitative analysis, utilizing inductive coding during the interviews.

The analysis of collected textual user feedback, prototype comparison, SUS score analysis and comparison, and qualitative expert interviews contribute to assessing the usability, effectiveness, and user satisfaction of We!Masomo, a web-based application aimed at promoting sexual health education in sub-Saharan Africa.

All findings from the Problem Room are consolidated in the subsequent study and available online through the web application We!Masomo [43].

### Ethical Considerations

The research adheres to the principles outlined in the Declaration of Helsinki and has received approval from the Institutional Review Board (or Ethics Committee) at Witten/Herdecke University (protocol code S-119/2022; approval date August 6, 2022). All participants in the study provided informed consent for publication before participation. Participants in the study signed a consent form after receiving written information and a verbal explanation about the study’s context, procedures, and data usage. Each participant received a copy of the signed informed consent documentation. To protect confidentiality, all study data were anonymized to ensure participants could not be identified in any publications or research data. Importantly, participants did not receive any financial compensation for their involvement in the study.

## Results

### Case Study Approach Within the Development and Delivery Phases

Analyzing insights from various methodology handbooks in the social science and information systems fields, we refined the low-fidelity prototype She!Masomo by addressing cultural differences and overcoming limitations to enhance future usability. This evolution led to the delivery phase, where we analyzed the high-fidelity prototype We!Masomo through 4 distinct analysis steps tailored to the specific needs of the software engineering process. The case study methodology is well suited for this approach and study because contemporary phenomena are difficult to study in isolation.

### Case Study Design Within the Development Phase: Using Textual User Feedback From the Low-Fidelity Prototype She!Masomo to Develop the High-Fidelity Prototype We!Masomo

The development phase resulted in the creation of a low-fidelity prototype named She!Masomo, along with an analysis of the end user’s environment. Moving into the delivery phase, we introduced the high-fidelity prototype, We!Masomo, specifically designed for sexual health education in sub-Saharan Africa. This version features expanded content and targets a broader audience, encompassing both women and men.

### Implementing Textual User Feedback—Adjustment of the Low-Fidelity Prototype She!Masomo in the Development Phase

In the initial phase of analysis, adolescent women tested the low-fidelity prototype to evaluate its accessibility, voice control, and features aimed at establishing a secure familial environment, as highlighted by Rau et al [23]. User feedback was collected through self-assessment (63/77) and analyzed using the affinity mapping technique. This process revealed 4 distinct clusters through inductive coding.

In cluster 1, 36/63 (57%) participants provided nonconcrete feedback on the low-fidelity prototype. Responses varied; some suggested that no additional features or content are necessary, while others expressed positive sentiments about She!Masomo, stating, “I believe it's a valuable system as it enhances our understanding of health.” One participant notably underscored the significance of women’s health, asserting, “No changes are needed; it is crucial for women to maintain good health as men often neglect their well-being.” Participants also conveyed that the tool is easy to use, anticipating that most individuals would quickly grasp its functionality. A forward-looking perspective was evident as well, highlighting, “This tool is secure and beneficial for girls and women in the community.” However, several participants noted the tool’s ease of use and advocated for broader outreach, suggesting its introduction in schools and community centers to effectively reach a wider audience, including men. They emphasized the importance of informing both men and women about health topics. One participant indicated the need to include content relevant to males.

Cluster 2 consisted of technical improvement suggestions, with 7/63 (11%) focusing on enhancing the platform’s interface, user-friendliness, and visual appeal. Recommendations included the introduction of a log-in page for user profile verification, a search bar for specific content suggestions, integration with social media, and implementation of robust privacy regulations.

Cluster 3 encompassed content suggestions, with 17/63 (27%) participants defining 3 key areas of emphasis: gender equality and violence; the inclusion of men in conversations; and information on cervical and breast cancer, parenting styles, menstruation, and sexual reproductive health rights. There was a notable demand within the realm of gender equality and violence to expand the content to include the male perspective. A participant expressed, “Violence and abuse against women - what can we do? What do we have to do initially? Can we talk?” Another participant emphasized, “It is very attractive to use, but the men’s content is missing. Also, men need to know this information.” One participant conveyed, “This tool has helped me with family planning. It makes my marriage peaceful and nice, so my husband is nice to me. It also helps me understand why I need to go to the hospital regularly to check and when to go and to learn and understand more.” Participants expressed a need for content related to cervical and breast cancer, as well as information on sexually transmitted infections. They emphasized the importance of being able to identify and conduct home testing when access to hospital visits is limited. Additionally, the subcluster focusing on parenting styles and family planning received considerable attention. One participant inquired, “How to give birth to a child and how to raise it?” They highlighted the necessity for guidelines on parenting styles; developmental milestones for children; what activities are appropriate at various ages; and when children typically achieve milestones such as walking, talking, and engaging in tasks. Participants requested comprehensive and easily understandable content, preferably in video format, available in both English and Swahili. They also asked for information on nutritional intake. Additionally, participants expressed the need for a subsection on menstruation. They sought information on how the monthly cycle functions, the physiological changes in the female body, and details related to menopause. This prompted requests for more informative videos, as well as general information on the contextual framework of sexual and reproductive health (SRH) rights and services, which often differ in East Africa. They also wanted to understand the cultural differences and asked for guidance on whom to speak to regarding this topic.

In cluster 4, improvements focused on potential feature extensions. Only 3/63 (5%) participants expressed a desire for additional features, such as a period tracker and a 24-hour online service for inquiries related to sexual well-being and emergencies. Additionally, participants emphasized the importance of user engagement through features that facilitate questions and discussions on controversial topics. Furthermore, incorporating a period tracker could serve as an optional contraceptive method, aligning with the extensive list of contraceptive methods. Another participant expressed a need for a 24-hour online service, allowing users to inquire about any matters related to sexual well-being and emergencies at any time. This indicates a preference for an optional hotline feature to be included in the platform. Notably, a participant underscored the importance of user engagement, expressing the desire for an additional feature that allows for questions and discussions. This functionality could be facilitated through an exchange platform for user interaction. The evaluation marks the transition from She!Masomo to We!Masomo. We!Masomo, the developed high-fidelity prototype, is coded via GitHub [44], deployed on Vercel (Vercel Inc.), with Google Firebase (Google LLC/Alphabet Inc.) as the back end. This concludes the development phase of the Solution Room of the Intercultural Research Model, as shown in Figure 1, and is followed by the delivery phase.

### Comparing Low- and High-Fidelity Prototypes in the Delivery Phase

Step 2 of the analysis involved continuous engagement with potential users over an extended period. The Intercultural Research Model process concluded with the delivery phase, where the previous results were synthesized into a solution [38]. These findings guided the further development of a high-fidelity prototype to refine the detailed concept. This process included considerations of sizes, fonts, colors, and content. The high-fidelity prototype integrates advanced functionalities and serves as the final minimum viable product [38]. In terms of intercultural design, these mechanisms will be evaluated for their effectiveness within the target culture. The primary focus is on Kenyan users, ensuring that features are tailored to meet identified needs as comprehensively as possible.

### Case Study Design Within the Delivery Phase: High-Fidelity Prototype We!Masomo—Introduction to the Web-Based Application

Based on the outcomes and feedback from the low-fidelity prototype, along with insights gathered from the SUS study [29] and textual user feedback, the development of the high-fidelity prototype as a web-based application with online user interactions has commenced. This advanced high-fidelity prototype, We!Masomo, serves as a visual representation of the user interface, integrating refined feedback. Figure 2 illustrates the freely accessible web-based application, We!Masomo [43] (screenshot taken on February 4, 2024), developed based on the structure of the low-fidelity prototype, She!Masomo. Table S1 in Multimedia Appendix 1 provides a full explanation of the requirements mentioned, including task appropriateness (TA), self-descriptiveness (SD), controllability (C), conformity to expectations (CE), learnability (L), and user retention (UR).

In accordance with requirement UR3, the tool is designed to be accessible without necessitating user log-in, outlining the application framework to ensure accessibility for all without requiring a user profile. The “Welcome” page introduces users to the tool, providing guidance on its optimal use and specifying the cookies that are tracked. Addressing user uncertainties regarding the sensitive content of the tool, we included an introductory text (L1) aimed at alleviating concerns (Figure 2). It advises users to consider their environment, recommending a quiet, undisturbed moment for private engagement with the content. To create a welcoming atmosphere and provide guidance, a fictional character named Linda, portrayed as a supportive big sister figure, is introduced. Per requirement UR4, the placeholder is positioned in the upper-left corner of the introduction box, as outlined in the storyboard of the primary version (see Figure S3 in Multimedia Appendix 1). Users are offered the option to close the introduction using a cross located in the upper-right corner. If they wish to revisit the introduction, they can click on “Help.” The start screen is designed to be accessible without log-in credentials, intentionally crafted to encourage exploratory experiences. This approach eliminates the immediate requirement for users to disclose personal information and is provided free of charge (TA3). Subsequently, users are guided through the tool by the fictional characters Linda for She!Masomo and Leo for He!Masomo. After the initial presentation of the start screen, the next page is the “Explore” page. Beginning from the main page, various navigation options are provided to enhance the exploratory experience of the application. The core of the application is its “Content” page, which features menu points directing users to specific thematic areas covered by We!Masomo. These include details on contraceptive methods, expanded content on cervical and breast cancer, menstruation, parenting styles, mental health issues, and myths (TA8). One of these menu points is contraception methods (C2), displayed on the “Content” page. Upon selecting a specific topic, users are directed to an overview of categories within that topic (C2). From this point, users can delve into further subcategories. For instance, in the structure of “Contraception Methods,” if subdivisions exist, a tab bar will appear, featuring these subcategories (C2). These subcategories are visually presented in the bar to cater to users with varying levels of alphabetical knowledge. The selected option is highlighted in color while the unselected ones are grayed out. Additionally, auditory and textual versions are provided (TA12). To enhance accessibility, especially for users with limited literacy, each thematic area includes graphics and voice output. The interactive design integrates both written and auditory formats for all text passages, enabling a read aloud function supported in English and Swahili. This functionality is demonstrated on the “Content” page. Strategically placed speaker icons activate text-to-speech functionality for label reading, improving user comfort, particularly for those with limited reading proficiency. Structurally, the “Content” page is composed of modules, each organized consistently for ease of navigation and comprehension. Each module begins with a header stating the topic of discussion, similar to the header section, followed by textual, visual, and auditory representations. An explanatory text of varying length is placed below these representations. The speaker icon, distinct from the buttons, is positioned beneath the text. Furthermore, each module provides information on how users can access more detailed and personalized information. During the content-scrolling process, both the header section and tab bar remain visible, enabling users to seamlessly switch between subcategories or return to the previous page (C1). The content is designed to convey the informative and professionally validated nature of the application (TA1 and TA4). At the bottom of the page, an upward arrow appears to facilitate users’ return to the top of the page (SD1). Additionally, there is a feedback option available, represented by a thumbs up or down (dislike) option. Giving feedback is a valuable way for users to indicate if improvements to the content are needed.

In addition to the start screen, each page includes a header section at the top, which features the logo, language selection options, and a hamburger icon for navigation. The language selection feature (CE2) enables users to choose their preferred language and remains visible throughout their entire user journey. It is streamlined with globe icons representing the English and Kenyan flags. The currently selected language is highlighted, while the unused language appears in gray. Both the hamburger icon and the logo icon serve navigation purposes within the app. The hamburger icon opens a menu, as explained in the following section, while the logo icon links back to the main page, which aligns with requirement C1 and allows users to apply their knowledge (SD1). In future development stages, graphic placeholders will be replaced with sketches to provide a more abstract representation, which will help mitigate potential embarrassment (CE5). By contrast, navigating using the Hamburger icon introduces an additional step compared with navigating via the main page (C1). As the icon is accessible from any page, this approach involves an extra step by displaying all menu items. When the Hamburger icon is selected, it changes color to blue to indicate the current selection, aligning with the language selection feature. Clicking on another area closes the menu, and the icon returns to its original color (SD1). As shown in Figure 2, a dedicated space for the We!Masomo logo (UR5) is included to inspire trust (CE6). In cases where user log-in is required, as depicted on the user log-in page, it is necessary to access newly developed features such as the exchange platform and period tracker, ensuring the privacy of users’ data.

Considering participants’ improvement suggestions from textual user feedback, the end user will be able to select their gender and indicate preferences for genders of interest. As a feature extension, in alignment with CE7, future development may include a health care center locator (TA15). This could potentially leverage tools such as Google Analytics (Google LLC/Alphabet Inc.) to enhance usability and accessibility. During the high-fidelity prototype development phase, the health care center locator feature was omitted. However, various other features have been successfully integrated into the prototype.

### SUS Data Analysis of the High-Fidelity Prototype in the Delivery Phase

Assessing We!Masomo using the SUS methodology, as outlined by Brooke [40], represents step 3 of the multimethod approach to measure the improved usability of the web-based application. This evaluation compares with the prior SUS score of 67 obtained from the low-fidelity prototype (She!Masomo). The comprehensive SUS survey involved 90 individuals and was conducted as part of the reevaluation during phase 4 of the Intercultural Research Model. The repeat of the SUS analysis for We!Masomo was conducted in the fourth phase of the Intercultural Research Model. Out of the total responses received (N=90), 82 (91%) responses using We!Masomo were considered complete and included in the subsequent analysis; 8 observations were excluded as a result of missing values. Descriptive characteristics of the participants from the LimeSurvey are detailed in Table S2 in Multimedia Appendix 1.

Conversely, for negatively worded questions, the score contribution is determined as 5 minus the scale position. The overall SUS score is calculated by multiplying the sum of the score contributions from each questionnaire by 2.5, following Brooke’s methodology [40]. An SUS score of 68 or higher is considered good [41]. The questionnaire details are illustrated in Table S3 in Multimedia Appendix 1. The SUS questionnaire consists of 10 questions, each rated on a 5-point Likert scale, following Brooke’s guidelines [40]. For positively framed questions (1, 3, 5, 7, and 9), the score contribution is calculated as the scale position minus 1. For negatively framed questions (2, 4, 6, 8, and 10), the score contribution is determined using reversed scale positions (5 minus scale position). Based on these calculations, the computed SUS score, placed on a scale from 0 to 100, is determined to be 77.3. Interpreting this score, which falls within the “high marginal” to “acceptable” range, it is classified as “good” based on adjective ratings. Acceptability scores typically rank above 70 as acceptable, below 50 as unacceptable, and scores in between as marginal [34,38]. However, it is crucial to note that not all requirements from the initial requirement engineering analysis were fully met or significantly adjusted during the development phase, as originally suggested based on feedback from the low-fidelity prototype. This suggests areas for potential improvement or further refinement in subsequent iterations of the application. Despite this, the high-fidelity prototype remains acceptable, fostering a positive behavioral intention to use. However, continuous improvement and adjustment are essential to meet the evolving needs of its users.

### Expert Interview—Review and Feedback on We!Masomo in the Delivery Phase

The 4 expert interviews, conducted as step 4 in the qualitative research methodologies, provide valuable insights into user feedback on the high-fidelity prototype, We!Masomo. These interviews highlight its superiority as a more user-friendly tool compared with She!Masomo. In the fourth and final analysis stage, these insights delve into user experiences, perceptions, and suggestions for further enhancements. Following Kuckartz’s [42] evaluation methodologies, qualitative structured content and inductive coding were applied during expert interviews, adhering to established qualitative research practices. This approach ensures systematic analysis and interpretation of the insights gathered from the interviews.

The main insights from the 4 expert interviews resulted in 105 Post-it notes categorized into 4 distinct clusters (see Figures S4 and S5 in Multimedia Appendix 1). These clusters offer a comprehensive understanding of various perspectives, including social belonging, education, technology aspects, and application insights. Notably, the experts emphasized the significance of We!Masomo as a digital educational application, particularly in addressing the challenges related to menstruation in sub-Saharan Africa and scaling knowledge and education effectively. They highlighted the complexities of societal norms, cultural beliefs, and educational barriers that impact sexual health awareness. Furthermore, the experts underscored the importance of technology accessibility, language inclusion, and creative engagement strategies in designing effective educational interventions. The experts provided perspectives from backgrounds in Kenyan social work and education.

In cluster 1, social belonging comprised participants’ characteristics from Kenyan nongovernmental organizations (NGOs), teachers, and social workers, offering diverse perspectives based on their backgrounds and experiences with end users. B1 (yellow) is a Kenyan male social worker and founder of a local information technology education campus in rural villages in northern Kenya. He expresses a supportive viewpoint, highlighting the significance of religion and culture, as well as the importance of empowering women and men through education. B2 (orange), from the slums around Nairobi, shared insights gained from working for a local NGO and founding a community center. His involvement in women’s empowerment groups and menstruation education adds depth to his perspectives. He has a daughter and oversees the technical aspects of the NGO. His perspective is shaped by interacting with users and his extensive experience in understanding the requirements needed to improve sexual health education. B3 (purple), a Kenyan start-up founder from Nairobi, previously worked for international development aid companies. She provides insights from the viewpoint of an educated urban dweller, where women have greater access to education. She established her business around a mentorship program, selling it to local partners and NGOs. Her mission is to mandate a mentorship program for all girls in secondary school to educate them about menstruation. B4 (green), originally from Nairobi, studied music at Nairobi University and founded her own NGO focusing on SRH and rights. Her diverse background, spanning Kenya and Denmark, equipped her to offer insights from an educated perspective, particularly regarding schooling systems and international education standards. This cluster highlighted the diverse social affiliations of the experts.

Cluster 2 focuses on education, addressing key aspects of the local schooling system, including reasons for school dropouts and the absence of sexual health education in the curriculum. In Kenya, most children are enrolled in school, benefiting from its free accessibility. However, secondary education typically requires fees, leading to a disparity where boys are more likely to attend secondary school compared with girls, as highlighted by B1, B2, and B3. The current government intends to increase school fees, which may lead to higher rates of school dropouts. Additionally, according to B2, most primary schools are not entirely free as school administrations still request money from parents. In certain regions, it remains uncommon for girls to pursue secondary education, as noted by B3. B3’s start-up focuses on menstruation education to encourage girls to stay in school. According to B3, only 30% of girls attend secondary school due to missing 1 week per month because of menstruation. Unfortunately, sexual education and contraceptive education are largely absent from secondary school curricula, as emphasized by B1, B2, and B3. B2 attributes the lack of comprehensive education to governmental policies and a conservative mindset. It is imperative to advocate for the inclusion of comprehensive sexual education in secondary school curricula, and initiatives such as We!Masomo could significantly contribute to this endeavor.

In cluster 3, focusing on technology aspects, B2 highlighted the divide between internet access in rural and urban areas. According to B1, smartphone and internet coverage reaches approximately 80% of the population in Kenya. Smartphone usage is increasing due to various initiatives, including government programs distributing smartphones in rural areas and support from Starlink (Starlink Services, LLC), which enhances accessibility. At B1’s NGO, every student receives a free computer to use and participate in the training program. B1 emphasizes that NGOs play a significant role in providing access to computers and internet facilities, especially in rural low-income areas. The widespread use of the Kenyan online payment system M-PESA ensures that most individuals already have a mobile phone, which facilitates digital transactions. M-PESA is a mobile money transfer and financial service for smartphones, owned by the local telecommunications provider Safaricom in Kenya. It allows users to deposit, withdraw, and transfer money, pay for goods and services, and access credit using their smartphones. M-PESA is widely used and deeply integrated into daily life, serving as a reference point for Kenya’s status as a digital pioneer. It has revolutionized financial inclusion in many parts of Africa by providing banking services to the unbanked population. B3 noted that at least one friend in every group in rural areas owns a smartphone. Additionally, schools, community centers, and NGOs provide computer rooms, which expand digital access. These factors underscore the potential reach and impact of a digital sexual health education application such as We!Masomo in urban areas across Kenya.

Cluster 4 highlights application insights, discussing improvement suggestions for We!Masomo. All 4 participants expressed strong support for We!Masomo and endorsed its introduction to their communities. They emphasized that while not everything needs to be included initially, the application serves as a foundational resource for learning. B1 emphasized how technology can motivate girls to pursue education, as he does at his NGO. B3 likened the application to a supportive elder sister, providing essential information about sexual health. Suggestions included incorporating more graphics and videos to enhance usability, as well as testimonials suggested by B2. Participants appreciated that everything was translated into Swahili and English, with audio files available. The expert interviews provided a nuanced understanding of user perceptions and preferences, affirming We!Masomo’s potential as a transformative tool.

The synthesized findings from expert interviews, along with other analytical components, inform continuous refinement and enhancement of We!Masomo to better serve its targeted audience and advance sexual health outcomes. The evaluation included textual user feedback, prototype comparisons, SUS analysis, and qualitative interviews, collectively contributing to assessing We!Masomo’s usability, effectiveness, and user satisfaction. Today, we have enrolled 250 user profiles in the educational digital tool.

## Discussion

### Principal Findings

The analysis focuses on a practical triangulation case study approach, highlighting the development of a concept for educational digital tools aimed at achieving cross-cultural usability. This initiative aims to address culturally sensitive topics in resource-poor regions of sub-Saharan Africa, particularly in Kenya. The developed concept, tailored using this triangulated case study method approach, addresses taboos and the lack of sexual education and knowledge among adolescents [5,6,45]. It aims to tackle challenges associated with limited access to education and digital infrastructure, emphasizing the need for accessible content on intimate topics while respecting cultural and religious differences. By following the research objectives, we aim to evaluate system usability as a measure of the fit between the low- and high-fidelity prototypes, marking the transition from the Problem Room to the Solution Room. Additionally, research objective 2 involves assessing the differences between She!Masomo and the newly developed all-inclusive platform We!Masomo.

Therefore, the study utilizes the developed Intercultural Research Model, emphasizing cultural considerations throughout the tool development process and practical implementation in real-life usage. The case study accompanies the transition from the low-fidelity prototype, She!Masomo, to the high-fidelity prototype, We!Masomo, which has been successfully implemented. The results affirm the effectiveness of the Intercultural Research Model in enhancing usability and facilitating product development for culturally diverse settings.

The research objectives focus on evaluating how well the requirements of the Intercultural Research Model support the development and implementation of culturally sensitive SRH educational tools under challenging circumstances. The target group is specifically defined based on the social affiliations and circumstances of Kenyan men and women [7,8].

The objectives aim to ensure that all established requirements are met for the final tool implementation. However, despite the thorough requirement engineering analysis conducted, it is recognized that not all requirements need to be fulfilled to achieve high usability. This prompts a reassessment of the framework, anticipating that a shortfall in meeting certain requirements will not adversely impact the measurement of usability, as assessed by the SUS score. Answering objective 1, the study reveals a significant improvement in usability and acceptance of the high-fidelity prototype throughout its development process, as evidenced by its SUS score of 77. This is compared with the low-fidelity prototype, She!Masomo, which scored 67 [29]. For instance, the tool is designed to be accessible without requiring user log-in. However, to ensure data privacy, additional features that involve sensitive information require users to log-in. Furthermore, We!Masomo has been adjusted to include a feedback option, highlighting the iterative nature of the development process and the importance of user input in refining content. It is proven that integrating user- and human-centered evaluation, enriched by cultural ergonomics derived from the established Double Diamond Model, inspired by Rau et al [23], Lachner et al [24], and Laws et al [25], is essential but not sufficient for a successful development and implementation approach. Constant field testing and engagement with end users are essential requirements, emphasizing user- and youth-friendly services. School and health educators also play a significant role in the implementation and usage of such tools, as they provide positive feedback on their usage in communities. The study by Decker et al [46] demonstrated technology issues affecting the implementation of SRH technologies aimed at improving adolescent health, such as increasing condom usage and knowledge. Health educators are often not well-versed in technology or SRH education, leading to challenges in educating youth due to insufficient information provided. The study results are consistent with these difficulties, highlighting technological issues during data collection, problems with Wi-Fi hotspots, users forgetting log-in details, or malfunctioning technology on smartphones. Conversely, positive responses demonstrate acceptance of online resources and consent for using the tool as a local reference service in addition to on-the-ground services. The study conducted by Ouma et al [6] demonstrated that despite ongoing international commitments to increase accessibility and improve knowledge of contraceptive methods in Kenya, adolescents participating in the study still express safety concerns about the side effects of contraceptive methods.

Therefore, We!Masomo utilizes requirement CE6 by introducing the fictional characters Linda and Leo, designed to guide adolescents of both genders and create a trustworthy atmosphere, as specified in requirement L1. Enhancing usability is achieved through the implementation of additional content, such as male-specific content, information on HIV, child nutrition, and cervical and breast cancer. This expansion aims to support increased usage and address concerns highlighted in the study (O2), while also potentially increasing the frequency of visits, aligning with research objective 2. A study conducted by McMahon et al [47] demonstrated the feasibility and increased acceptability of eHealth interventions focusing on HIV content, while also offering insights into health-seeking behaviors [48]. It became evident that despite facing challenges in resource-poor regions, financial constraints, and cultural stigmatization, there is a pressing need for policy makers to adopt a variety of educational approaches to provide comprehensive SRH education to youth. Utilizing mass media for disseminating information has the potential to enhance awareness and utilization of modern contraceptives. Implementing similar initiatives focusing on segments of the population with low contraceptive uptake is advisable [49].

Looking ahead, future studies should involve a broader range of health and school educators in the evaluation process to help disseminate the tool and educate them about its usage. Local educators play a crucial role in determining accessibility and identifying potential limitations of the tool. Additionally, it is important to include the public sector and policy makers to ensure practical implementation of We!Masomo. Furthermore, exploring the transferability of the Intercultural Research Model to other cultural contexts and considering demographics such as men who have sex with men will be critical for comprehensive research efforts [50]. Moreover, beyond research studies, the tool is intended for long-term use, necessitating considerations for UR and engagement without requiring frequent relogging or intervention usage. Despite the study conducted by Norton and Tappis [51], there is an opportunity for a standardized research model to better inform the evidence base for improving the delivery of SRH interventions in humanitarian settings. Using this self-adjusted Intercultural Research Model, practical use cases should be implemented and analyzed more closely through case study examples in the future.

It is important to acknowledge potential limitations and biases related to the sensitive nature of the topic, as well as biases among participants when addressing intimate topics considered taboo in society. These factors may lead to feelings of shame and discomfort, compounded by language barriers that can make detailed statements challenging to analyze [52]. It is important to note that the applicability of the study’s findings on digital tools for sexual health information is restricted to the specifically developed web-based application prototype, We!Masomo, intended for practical and easy-to-use implementation. Furthermore, caution should be exercised when generalizing the results, as a convenience sample was used, which may not accurately represent the broader population. This limitation arises from restricted access to the internet and devices, as well as varying levels of digital literacy. To improve comparability with other studies, future research should include standardized measurements such as the widely accepted SUS measurement, alongside context-specific methods [38].

### Conclusions

Individuals grappling with reproductive and sexual health challenges frequently confront educational disparities. Discussions on sexuality are often constrained by cultural stigmas and insufficient resources, yet sexual autonomy remains pivotal for well-being. Regrettably, data from 57 countries reveal that approximately 45% of women aged 15-49 in relationships or marriages lack this autonomy, particularly prevalent in sub-Saharan Africa [1]. This study aims to develop a reusable universal framework for creating a practical, scalable digital educational platform that addresses taboo and intimate topics. Specifically designed for resource-poor regions, this platform utilizes the concept of the Intercultural Research Model. This model, derived from the user-centered Double Diamond Model, serves as a guiding framework for the development process, emphasizing cultural considerations throughout. The study focuses on 14-35-year-old Kenyans as a case study, with the objective of providing accessible sexual health education that caters to the specific needs of both men and women in Kenya. The research methodology involves engaging with the targeted culture and reassessing the digital tools used to deliver educational sexual health information effectively. Requirement engineering analysis suggests enhancing text communication with visual and auditory channels. This includes incorporating fictional characters to guide users through the digital tool, aiming to reduce taboos and overcome language barriers. Additionally, features such as user log-in, an exchange platform between users, and feedback options are recommended. The study proposes conducting on-site evaluations of both low- and high-fidelity prototypes to explore interactive user feedback effectively. A triangulated multimethod approach, combining textual user feedback collection, prototype comparison, SUS analysis on We!Masomo, and comparing SUS scores with the low-fidelity prototype, alongside qualitative expert interviews, highlights the importance of accessible intimate content while respecting cultural and religious differences [31]. The findings emphasize the ongoing necessity to refine and explore methods to ensure inclusivity and effectiveness across diverse cultural contexts. The study’s objectives, which include evaluating system usability and discerning differences between prototypes, underscore the critical aspects of the Solution Room, spanning both the development and delivery phases. This process culminates in the creation of the high-fidelity prototype, We!Masomo. The primary goal of We!Masomo is to offer free digital information on contraceptive methods and menstruation education, with the aim of empowering and educating users, especially in regions where access to sexual health knowledge is limited. Social stigmas, cultural nuances, and religious beliefs have been identified as substantial barriers to sexual health knowledge, underscoring the need for tailored educational content that addresses specific circumstances. This discussion emphasizes the complex interplay between cultural considerations, usability evaluation, and existing research in the field. It stresses the significance of adaptive methodologies capable of accommodating diverse contexts while adhering to user-centered design principles. Looking ahead, there is consideration for expanding We!Masomo to other regions, despite not meeting all requirements of the self-established Intercultural Research Model. The digital tool, We!Masomo, shows a high behavioral intention to use, as indicated by its SUS score and successful implementation. In conclusion, this study underscores the substantial progress achieved in tackling reproductive and sexual health challenges using digital educational tools. The tailored survey methods highlight the potential effectiveness of adjusting the initially suggested requirements for successful implementation. This opens avenues for exploring the impact of cultural and religious factors on technology acceptance and paves the way for reaching a larger segment of the population in the future.

## Appendix

Multimedia Appendix 1 Details on the case study, approach, and results.

Multimedia Appendix 2 Survey questionnaire.

## Abbreviations

CHERRIES: Checklist for Reporting Results of Internet E-Surveys
COREQ: Consolidated Criteria for Reporting Qualitative Research
NGO: nongovernmental organization
SRH: Sexual and reproductive health
SUS: System Usability Scale

## Data Availability

The data sets generated and analyzed during this study are available from the corresponding author on reasonable request and under consideration of the ethics committee’s requirement to maintain anonymity.
