# Supplementary material for: A Digital Sexual Health Education Web Application for Resource-Poor Regions in Kenya: Implementation-Oriented Case Study Using the Intercultural Research Model
Source: JMIR Form Res. 2024 Jul 3;8:e58549. doi: 10.2196/58549 (PMC11255522; doi:10.2196/58549)
Supplement: Multimedia Appendix 1 [file formative_v8i1e58549_app1.docx]

# Multimedia Appendix

*Figure S1: User-centered Double Diamond approach*


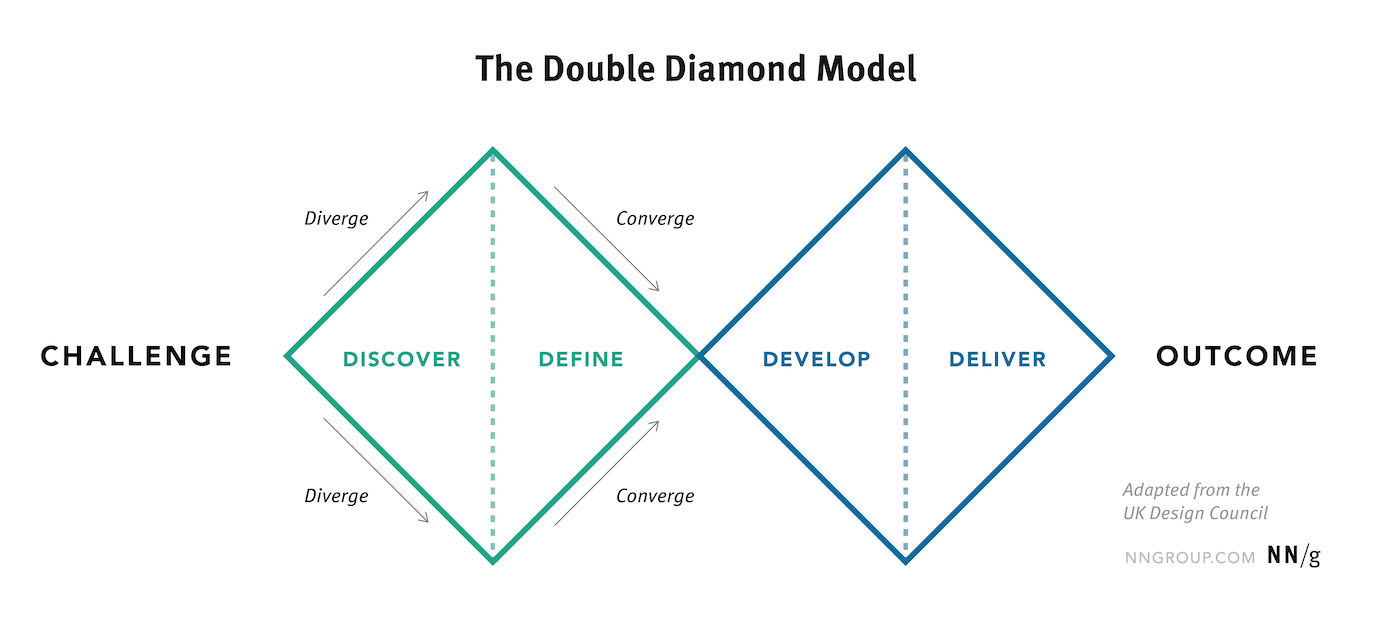


Source: UK Design Council, Nielsen Norman Group

Figure S2 - Intercultural Research Model, own illustration based on the Nielsen Norman Group Double Diamond Model and Rau et.al (2013)

Paper 1: Creating an intercultural user-centric design for a digital sexual health education tool for young women in resource-poor regions of Kenya: A Qualitative self-extended Double Diamond Model for Requirement Engineering Analysis ^14^ and Paper 2: An acceptance analysis of a sexual health education digital tool in resource-poor regions of Kenya: an UTAUT based survey study ^15^

Textual User Feedback

SUS

WE!Masomo High-Fidelity Prototype

Implementation

Interviews

Compare Study

UTAUT

She!Masomo Low-Fidelity Click Dumm Development

SUS

***Discover***

***Define***

***Develop***

***Deliver***

Paper 1,

**Problem**

**Problem Room**

Problem Statement

Interviews

Storyboard

Persona

Empathy Maps

**Solution Room**

Paper 2

Requirement Engineering Analysis

### *Figure S3 - Primary Persona – Ivy*


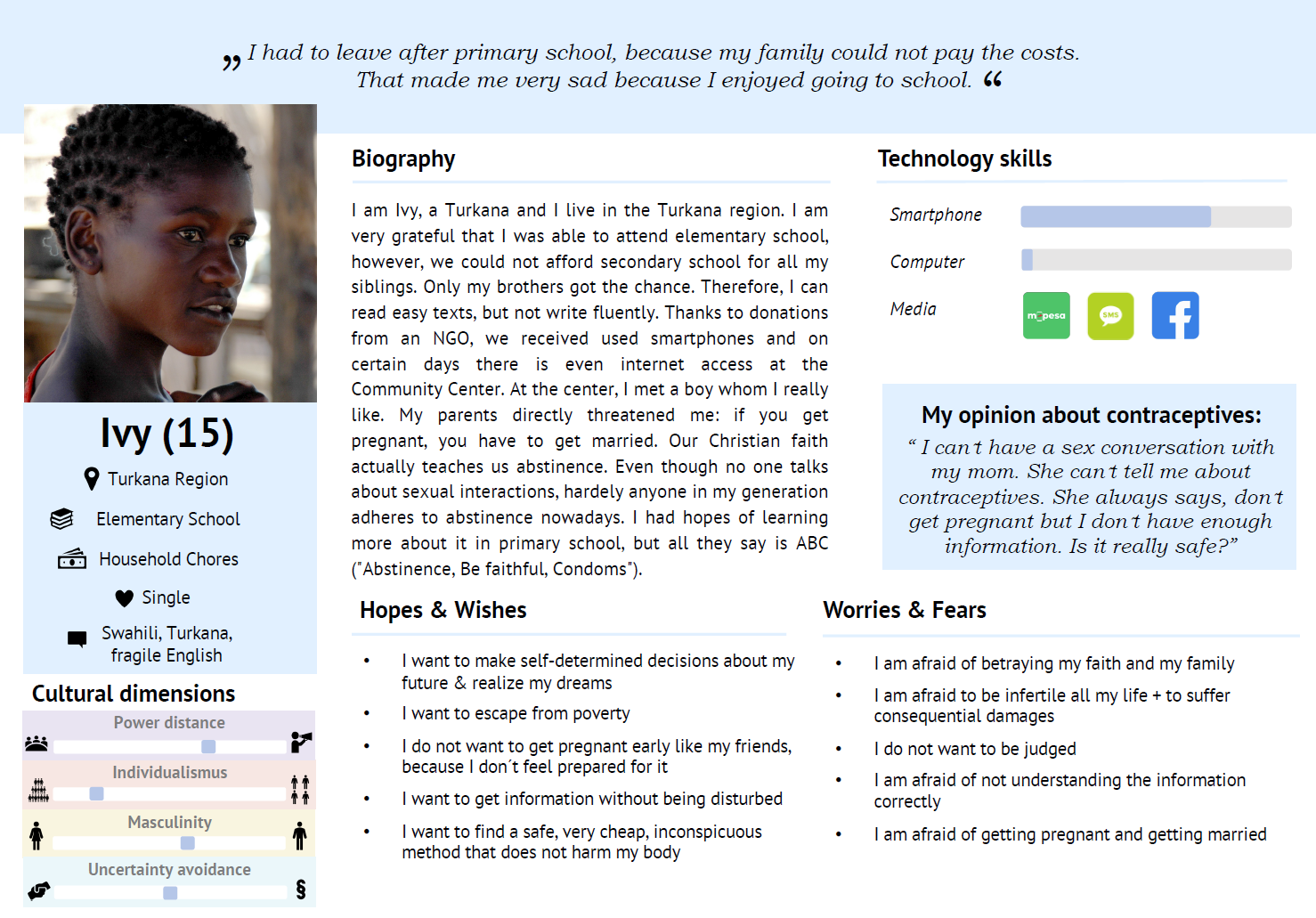


Source: Own illustration

Figure S4 – Ivy - Story Board


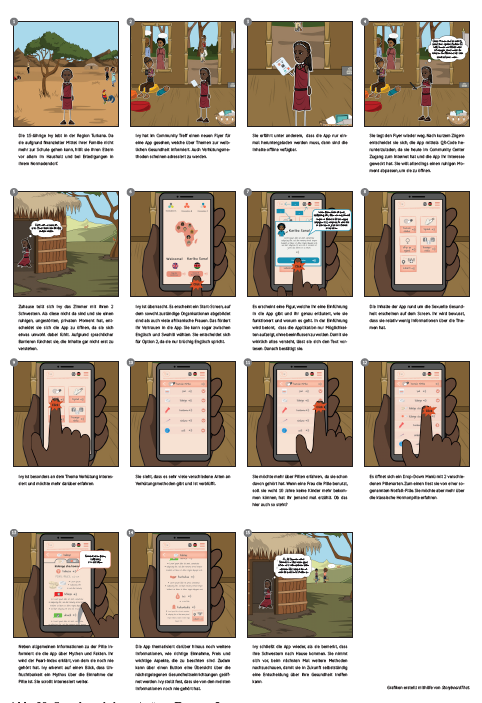


Source: Own illustration with StoryboardThat (Clever Prototypes, 2022)

Figure S5 – Expert Interview Post-its


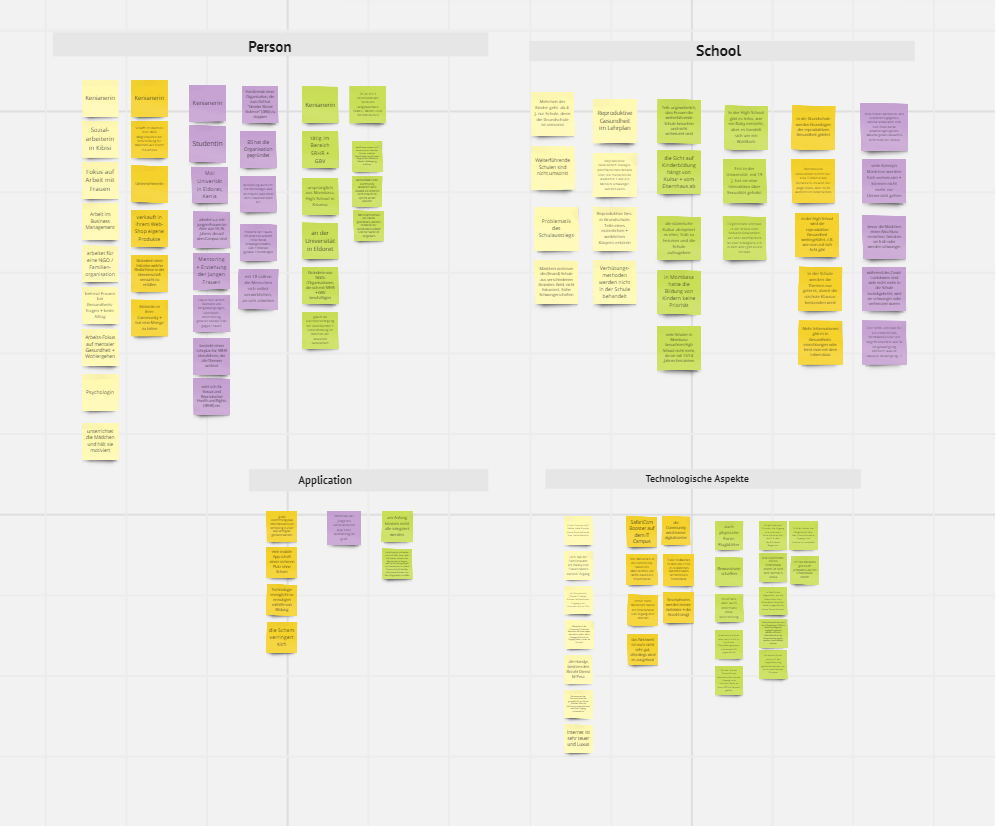


Source: Own illustration

Table S1 – Requirements Engineering Analysis

| Task appropriateness  „An interactive system is task-appropriate if it supports users in completing their tasks, i. that is, when the operating functions and the user-system interactions are based on the notable properties of the task.“ [33]  In an intercultural context: The expectations of the users should be met and lead them to their goal [31]. Information that is not suitable for the users can be stressful [33]. Accordingly, the application is intended to support users in dealing with such a sensitive topic by reducing uncertainties and answering questions. | | | |
| --- | --- | --- | --- |
| ID | TYPE | REQUIREMENT | PRIORITY |
| TA1 | NF | The application has a purely informational character, without consciously influencing users. |  |
| TA2 | NF | All content of the application is available offline after downloading. |  |
| TA3 | NF | The application can be accessed free of charge at any time. |  |
| TA4 | NF | The contents of the menu item "Contraceptive methods" are provided and checked by health professionals; they provide scientifically well-founded information. The indication that the information is checked by specialists appears under the textboxes. |  |
| TA5 | FA | Under the menu item "Contraceptive methods" for each form of contraception an introductory text with a short description is shown, and it is explicitly mentioned that contraceptive methods prevent pregnancy when used correctly. |  |
| TA6 | FA | Under the menu item "Contraceptive methods”, category condoms, the introduction describes that condoms can be used to prevent pregnancies as well as to prevent venereal diseases. |  |
| TA7 | FA | Under the menu item "Contraceptive methods" appears for each contraceptive method and textbox with information for taking and how to apply each method. |  |
| TA8 | FA | Under the menu item “Contraceptive methods”, a textbox “Myths and facts” for every form of birth control, to eliminate the misconceptions of the users. |  |
| TA9 | FA | In the menu item "Contraceptive methods" a textbox appears with information concerning security for every form of contraception. |  |
| TA10 | FA | Under the menu item "Contraceptive methods" an information box with price indications appear of the respective form of contraception. |  |
| TA11 | FA | Under the menu item “Contraceptive methods”, advantages and disadvantages of each form of contraception are listed. |  |
| TA12 | FA | Under the menu item "Contraceptive methods" an impact score of each contraception method is shown | X |
| TA13 | FA | Under the menu item "Contraceptive methods: Pill" an area appears which explains the different provider and the limited side effects when using different pill brands. |  |
| TA14 | FA | Under the menu item "Contraceptive methods" appears, for each contraceptive method, the indication that in health care facilities more detailed information can be provided to the user, as well as with the hint that the application cannot replace a specialist. There is also in the menu an item called "Healthcare facilities", locating different facilities. | X |
|  |  | Health facilities menu item |  |
| TA15 | FA | In the menu item "Healthcare facilities" the users can check whether condoms are free of charge available in the respective facility. | X |
| Self-descriptiveness  “Wherever required by the user, the interactive system provides appropriate information that make the capabilities of the system and its use immediately apparent, without that this requires unnecessary user-system interactions." [33]  In an intercultural context: The application should be always clear and understandable navigate [31]. Due to the limitation of the target group, a certain affinity for technology can be assumed. It is important to have well-known mental models’ users so that they can recognize them in the application. | | | |
| ID | TYPE | REQUIREMENT | PRIORITY |
| SD1 | NF | Mental models are being used within the application (e.g., the hamburger icon). |  |
| Controllability  “The interactive system allows the user to take control of the user interface and retain the interactions, including speed, sequence, and customization of user-system interaction." [33]  In an intercultural context: Due to the exploratory character, it is essential for the user to control the application according to their needs. This means for example the user can always get to the previous page [31]. | | | |
| ID | TYPE | REQUIREMENT | PRIORITY |
| C1 | FA | A "back arrow" can be used, and the logo icon and the Hamburger icon navigate through the application. | X |
| C2 | FA | The menu item "Contraceptive methods" is divided into further subcategories, from which the users reach each method (e.g., contraceptive methods à pill/ hormone-based pill & emergency pill) | X |
| C3 | Fa | As part of the application, a menu item called "Healthcare facilities" is given. It shows health facilities, surrounding the users depending on the location. | X |
| Conformity to Expectations  “The behavior of the interactive system is predictable based on the context of use and generally recognized conventions in this context." [33]  In an intercultural context: Even if the application confronts users with a taboo confronted topic, they should not feel overwhelmed. Every menu item should not contain any surprising information and should correspond to existing content of the users knowledge and experience [31]. | | | |
| ID | TYPE | REQUIREMENT | PRIORITY |
| CE1 | NF | Technical terms are explained to the users in simplified form. | X |
| CE2 | NF | The application is supported in ​​English and Swahili. | X |
| CE3 | NF | All text passages can be listed to via a read out loud function (speaker symbol). | X |
| CE4 | NF | In the application, text inputs are being avoided due to knowledge barriers. Necessary text entries (e.g., when entering birthday) are possible via a voice function (verbal support). | X |
| CE5 | NF | Every menu item, every birth control method and every headline are illustrated in a sketch-like representation, in addition to the text (visual support). | X |
| CE6 | FA | The logos of the participating organizations or NGOs are included with a link to the organizational webpage. | X |
| CE7 | FA | As part of the application, a menu item "Organizations" is listed, which directs users to Kenyan social Media platforms related to sexual health. |  |
| Learnability  "The interactive system supports the discovery of the abilities and their use, it allows the interactive system to be explored ('tried out'), minimizes the learning effort and offers support when learning is required." [33]  In an intercultural context: Since the users may have a different know-how on how to use a mobile application, they should be supported in how to use it so that they can focus on the content [31]. | | | |
| ID | TYPE | REQUIREMENT | PRIORITY |
| L1 | FA | The users get an introduction when registering, during which the user learns about the content and the different functions of the application. The introduction is informed, and the users is advised to take a quiet and undisturbed moment to read the content. | X |
| User retention  “The interactive system presents functions and information in an inviting and motivating manner, and thus promotes continuous interaction with the system." [33]  In an intercultural context: The system should create trust [33] and thus promote the metaphor of a safe place. | | | |
| ID | TYPE | REQUIREMENT | PRIORITY |
| UR1 | NF | Within the application no religious, traditional, and tribal affiliations are being expressed. |  |
| UR2 | NF | A personal atmosphere is created by using pronouns like "I" and "you" are used. |  |
| UR3 | NF | The application can be used without logging in. This allows the users a feeling of safe place, without the feeling of judgement and exploration. | X |
| UR4 | FA | In the introduction, a fictional character appears (Linda and Leo) which are used as metaphor as the big sister or brother. | X |
| UR5 | FA | The logos appear on the start page of the application organizations or NGOs involved in the market launch, whereby the social impact character is emphasized. | X |
| UR6 | FA | Several African women appear on the screen at the homepage of the application to enable a sense of connection. | X |

Source: Own illustration

Table S2 – Description Sample Characteristics of the We!Masomo SUS Analysis

| Characteristics in Percentage (Frequency) | | | | | |
| --- | --- | --- | --- | --- | --- |
| Gender | | | | | |
| Female | | | **Male** | | |
| 70.7% (58) | | | 29.3% (24) | | |
| Location | | | | | |
| Kenya | | | **Another African Country** | | |
| 85.36% (70) | | | 14.6% (12) | | |
| Age | | | | | |
| Below 20 Years | | | **20 -30 Years** | | **Above 30** |
| 30.5% (25) | | | 59.8% (49) | | 9.8% (8) |
| Working Status | | | | | |
| Student | **Unemployed** | | **Working** | | **Others** |
| 30.5% (25) | 32.5% (25) | | 26% (20) | | 5.2% (4) |
| Education Level | | | | | |
| Primary  Education | **Secondary Education** | **Post-Secondary Education** | | **Tertiary**  **Education** | **Prefer not to answer** |
| 17.1% (14) | 42.7% (35) | 15.9% (13) | | 24.4% (2s0) | 1.3% (1) |
| Relationship Status | | | | | |
| Married | **In a relationship,**  **not married** | | **Not in a**  **relationship** | | **Others or prefer not to answer** |
| 8.5% (7) | 28.0% (23) | | 45.1% (37) | | 18.3% (15) |
| Digital Literacy | | | | | |
| Excellent | **Good** | | **Average** | | **Poor** |
| 26.8% (22) | 34.1% (28) | | 26.8% (22) | | 12.2% (10) |

Source: Own illustration

Table S3 - Questionnaire of the survey

| Section A | |
| --- | --- |
| Question English | Question Swahili |
| My gender is ... | Jinsia yangu ni ... |
| Where are you located? | Mahali uliko? |
| What is your year of birth? | Mwaka wa kuzali? |
| What is your current working status? | Hali yako ya ajira? |
| What is your highest educational level? | Kiwango chako cha juu cha elimu? |
| What is your relationship status? | Hali ya Uhusiano wako ni gani? |
| Do you own any of the following items?  Computer; Smartphone, Tablet, None | Je, unamiliki mojawapo ya hivi vifaa?  Tarakilishi; Simu ya rununu ya ‘Android’; Simu ya Tabuleti; La |
| If you want to get specific information. How often do you ...  Search online for information; Search online for health information; Search online for sexual health information | Ikiwa unataka kupata habari maalum. Ni mara ngapi wewe...  kutafuta ujumbe kwa mtandao; tafuta ujumbe wa afya mtandaoni; kutafuta ujumbe wa afya ya kujumuiana kingono mtandaoni |
| Please rate your digital experience. | Je, matumizi yako ya kidijitali ni mazuri kwa kiasi gani? Tafadhali pima utajriba wako wa ubunifu wa kisasa. |
| Section B - SUS |  |
| Question English | Question Swahili |
| I think that I would like to use this system frequently. | Nadhani ningependa kutumia hii mbinu mara kwa mara. |
| I found the system unnecessarily complex. | Nilipata mbinu hii kuwa ngumu kiasi. |
| I thought the system was easy to use. | Nadhani mbinu hii ni rahisi kutumia. |
| I think that I would need the support of a technical person to be able to use this system. | Nadhani nitahitaji msaada wa mtaalam mwenye ujuzi ili niweze kutumia kifaa hiki. |
| I found the various functions in this system were well integrated. | Nilipata sehemu mbali mbali za mbinu hii zimeunganishwa vyema. |
| I thought there was too much inconsistency in this system. | Nafikiri kuna sehemu ambazo sio sahihi kwa mbinu hii. |
| I would imagine that most people would learn to use the system very quickly. | Nikidhani, watu wengi wanaweza kujifunza kutumia mbinu hii kwa wepesi. |
| I found the system very cumbersome to use. | Nilipata mbinu hii kuwa ya kuchosha kutumia. |

Source: Own illustration
