# Supplementary material for: A Digital Sexual Health Education Web Application for Resource-Poor Regions in Kenya: Implementation-Oriented Case Study Using the Intercultural Research Model
Source: JMIR Form Res. 2024 Jul 3;8:e58549. doi: 10.2196/58549 (PMC11255522; doi:10.2196/58549)
Supplement: Multimedia Appendix 2 [file formative_v8i1e58549_app2.docx]

**Appendix B**

Table 1- CHERRIES for used web survey

| **Item Category** | **Checklist Item** | **Explanation** |
| --- | --- | --- |
| **Design** | Describe survey design | The target population are primarily young women in Kenya. Therefore we placed the study in community centers, girl schools and universities in Kenya. The sample is not a convenience sample. |
| **IRB (Institutional Review Board) approval and informed consent process** | IRB approval | The ethic committee of the university of Witten/Herdecke approved the survey on 06.08.2022 under the protocol code: S-119/2022. |
|  | Informed consent | Participants were informed on the welcome page of the survey. The Information and Consent for Participation in Research Study have been provided in English and Swahili. The participants in the study remain anonymous. By clicking the checkbox consent was confirmed. |
|  | Data protection | The survey was hosted and all data were stored on its own secure server. No personal information was linked to survey results in any way. |
| **Development and pre-testing** | Development and testing | The used methods (SUS) consists of mainly standardised questions, which have been proved in various previous studies. Pre-testing was conducted using a sample of women in the community center in Kenya as well as women in Germany. |
| **Recruitment process and description of the sample having access to the questionnaire** | Open survey versus closed survey | The survey was an open survey. |
|  | Contact mode | The study was placed in community centres, girl schools and universities in Kenya. This was supported by the social initiative of Boehringer Ingelheim, Making More Health, (MHH). The participants were able to share the link to the study with friends e.g. via WhatsApp, Instagram and Facebook. |
|  | Advertising the survey | The study was announced by the contact persons in the community centres, girl schools and universities. |
| **Survey administration** | Web/E-mail | The survey was hosted on its own web server by the University Witten/Herdecke in Germany, using the software LimeSurvey. |
|  | Context | The landing page of the survey was publicly accessible and distributed through an URL. This ensured that participants were able to share the survey. |
|  | Mandatory/voluntary | The survey was completely voluntary. Users could  access the landing page without completing the survey. |
|  | Incentives | No incentives were offered to participants. |
|  | Time/Date | The survey period was from 15.11.23 to 31.12.23. |
|  | Randomization of items or questionnaires | Survey items were not randomized. |
|  | Adaptive questioning | No adaptive questioning was used. |
|  | Number of Items | Section A: 9 questions  Section B: 10 standardized questions |
|  | Number of screens (pages) | One welcome page and 9 pages with survey items |
|  | Completeness check | Most survey items were mandatory, and respondents were prompted to complete outstanding items before leaving the survey page. |
|  | Review step | Participants were able to review and change their answers by clicking the Back button. |
| **Response rates** | Unique site visitor | No cookies or IP controls were used to ensure that people could participate consecutively from the same device. Participation devices were brought to the survey. |
|  | View rate (Ratio of unique survey visitors/unique site visitors) | Not measured. |
|  | Participation rate (Ratio of unique visitors who agreed to participate/unique first survey page visitors) | Not measured. |
|  | Completion rate (Ratio of users who finished the survey/users who agreed to participate) | Section A: 82/82 = 100%  Section B: 79/82= ~96% |
| **Preventing multiple entries from the same individual** | Cookies used | No Cookies were used. |
|  | IP check | No cookies or IP controls were used. |
|  | Log file analysis | Indicate whether other techniques to analyze the log file for identification of multiple entries were used. If so, please describe. |
|  | Registration | Not necessary, since the survey was an open survey. |
| **Analysis** | Handling of incomplete questionnaires | Only completed questionnaires were included in the analysis. |
|  | Questionnaires submitted with an atypical timestamp | Not used. |
|  | Statistical correction | No statistical correction procedures or weightings were used in the analysis. |

Eysenbach G: Improving the Quality of Web Surveys: The Checklist for Reporting Results of Internet E-Surveys (CHERRIES). J Med Internet Res 2004;6(3):e34. URL: <https://www.jmir.org/2004/3/e34>. DOI: 10.2196/jmir.6.3.e34
